# Risk Assessment on Continued Public Health Threats: Evidence from China’s Stock Market

**DOI:** 10.3390/ijerph17207682

**Published:** 2020-10-21

**Authors:** Junjian Gu

**Affiliations:** Faculty of Business Sciences, University of Tsukuba, 3-29-1 Otsuka, Bunkyo-ku, Tokyo 112-0012, Japan; gu.junjian.fw@u.tsukuba.ac.jp

**Keywords:** COVID-19, continued increasing threat, market reaction, provincial environment, China

## Abstract

Given the disturbing effects of the coronavirus disease 2019 (COVID-19) outbreak, we are motivated to examine whether the continued increase of the provincial public health threats affects the firms’ accumulative abnormal return. Using the 178,805 firm-day observations from Chinese listed firms from 10 January to 31 March 2020, we find that the accumulative abnormal return is significantly lower among firms located in the provinces where face the continued increase of new confirmed COVID-19 cases. The relations remain constant after several robustness tests. These findings suggest that investors concern about the potential risk when firms are located in the provinces with higher threats to public health. We also find that the negative effect of increasing public health threats on abnormal return is weaker for firms surrounded by a provincial environment with stronger information accessibility and economic growth. Overall, this study extends the literature by presenting systematic evidence on the effect of the continued increase of provincial public health threats on the market reaction in Chinese listed firms.

## 1. Introduction

The prior study shows that coronavirus-related research mainly focuses on virology, immunology, epidemiology, and so forth. However, there are few studies that discuss the risk assessment on the coronavirus disease 2019 (COVID-19) [1]. Understanding how investors engage in risk assessment on the COVID-19 outbreak is an essential issue because it related to the financial market performance and economic development. While previous studies have shown that coronavirus outbreak has a negative short-term impact on the stock market [2,3,4,5,6], and stock markets’ decline to be mainly affected by the news attention [7], no prior research has examined the question of whether and how investors view continued increasing regional public health threats from a large sample of daily COVID-19 disclosure information. To fill this gap, we perform comprehensive analyses on the association between continued increasing provincial public health threats and firms’ market reaction based on the Chinese listed firms. 

We focus on the Chinese market for several reasons. First, the earliest human cases of COVID-19 were reported in China in December 2019 [8]. The sample period in this study is from 10 January to 31 March 2020, which shows the early evidence on the effect of continued increasing public health threats, driven by COVID-19, on the stock market. Second, China is the largest emerging market and the second-largest economy in the world, and it plays an increasingly significant role in globalization. Third, the institutional development across provinces in China is uneven, which enhance the statistical power of tests on the province level effects. Overall, the research on the Chinese market reaction to the COVID-19 outbreak has profound implications for many interested parties from different countries.

We expect that firms located in the provinces where face continued increase of public health threats are more likely to have a poor stock market performance. Three reasons support our conjecture. First, the continued increase in the amount of new confirmed cases in one specific provincial region will enhance the uncertainty of the firms’ short-term and long-term performance in this area, which negatively influences investor valuations of local firms. Second, the continued increase of public health threats will enhance the local economic cost and then enhance the investors’ risk assessment. Third, the continued increase in the amount of new confirmed cases in one specific provincial region may increase the event risk, and investors would be less likely to hold the financial assets from that area. 

Of course, continued increasing provincial public health threats may not affect the local firms’ market performance. First, long-term investors might not be aware of the risks of the continued increase of COVID-19 cases. Second, the COVID-19 outbreak may bring firms opportunities to generate more products to meet the increased demand. Third, investors might not focus on the daily based non-financial information, which leads to less value relevance for the COVID-19 disclosure. These possibilities create tension to our research question and, thus, whether continued increasing provincial public health threats reduce local firms’ cumulative abnormal return is an empirical question.

We empirically examine the relationship between continued increasing public health threats and firms’ stock market performance using the 178,805 firm-day observations from Chinese listed firms from 10 January to 31 March 2020. We use continued increasing of provincial new COVID-19 cases to capture the increasing threat and use the short-window abnormal return measures to capture investors’ risk assessment of expected costs of the continued increasing threats. Consistent with our hypothesis, we find that, compared with firms located in the province where does not face increasing public health threats, the firms surrounded by continued increasing threats have a lower level of accumulative abnormal return. It indicates that the investor’s concern about the potential risk when firms are located in the provinces with higher threats to public health. 

In addition, the main result is robust to alternative research designs. First, we apply alternative measures of provincial public health threats and obtain consistent inferences. Second, we add a falsification test by generating a pseudo-threat. We do not find a significant relation between abnormal return and pseudo-threat, which strengthens our inferences. Third, we address endogeneity concerns by applying an instrumental variable approach. Specifically, following prior related research [9,10], we use immigrant ratio and emigrant ratio as instruments. Our inferences keep unchanged.

Moreover, we conduct two sets of cross-sectional analyses for corroborating the inference from the main tests. First, we conjecture that stronger provincial information accessibility will decrease the investors’ risk assessment by mitigating the information asymmetry. We expect a negative moderate effect of information asymmetry, proxied by the higher websites rate, media coverage, and mobile internet rate, on the relationship between increasing threats and abnormal return. Second, we assume that stronger provincial economic growth will decrease the investors’ risk assessment by enhancing the likelihood to have a positive outlook on the future economy. Thus, we expect that the negative effect of increasing threats on market reaction is less pronounced when the provincial economic growth is stronger, proxied for by the higher gross regional product rate, employment rate, and urbanization. Overall, the results of cross-sectional analyses support our conjectures.

To provide additional insights, we further conduct several additional tests. First, we expect and find that continued decrease of provincial public health threats is positively related to the accumulative abnormal return. Second, regarding the community lockdown, we find that the community lockdown mitigates the relationship between continued increasing public health threats and accumulative abnormal return. Third, we additionally examine the firm heterogeneity in the effect of increasing threats on market reaction and find that the negative effect of increasing threats is weaker for firms with a higher level of geographical diversification, operating cash flow, and with a clean audit opinion. Fourth, we find that our results are not driven by the fluctuations in the number of new confirmed COVID-19 cases.

Our studies contribute to the literature by examining the impact of continued increasing provincial public health threats on the local firms’ market performance. This examination is important because it sheds light on how firms’ market reaction affected by the regional change in COVID-19 cases. Unlike prior studies that test the market reaction at the stock market level, this paper provides evidence that continued increasing regional threats can enhance the event risk and environmental uncertainty and that investors’ risk assessment can reduce the local firms’ accumulative abnormal return. Moreover, this paper complements studies on the impact of the COVID-19 outbreak on the market reaction by focusing on moderate effects of province-specific factors. Specifically, we provide new insight into which provincial factors mitigate the negative effect of continued increasing public health threats on firms’ market performance (e.g., the provinces characterized by stronger information accessibility and economic growth).

The remainder of the paper is organized as follows. Section 2 discusses the background, literature review, and hypothesis development. Section 3 presents the sample selection, empirical model, and descriptive statistics. Section 4 illustrates the main results and robustness tests. Section 5 shows the results of cross-sectional analyses. Section 6 adds additional analyses and sensitivity checks. Section 7 provides conclusions and discussion. 

## 2. Background, Literature, and Hypothesis Development 

### 2.1. Background of COVID-19

World Health Organization (WHO) first released the novel coronavirus (2019-nCoV) situation report on 21 January 2020 and clarified the first human cases of COVID-19 were reported in Wuhan, China, in December 2019 [8]. The report also confirmed the initial 282 cases worldwide by 20 January 2020, which contains 278 cases from China, 1 case from Japan, 1 case from the Republic of Korea, and 2 cases from Thailand. In China, the cases were mainly confirmed in the Hubei Province (258 cases), then 14 cases in the Guangdong Province, 5 cases in Beijing Municipality, and 1 case in Shanghai Municipality [8]. On 23 January 2020, restrictions on mobility were imposed on Wuhan city, and partial movement restrictions were enacted in numerous cities across China. Prior studies show the positive effect of restriction of human mobility on the mitigation of the COVID-19 spread in China [9,10,11,12]. COVID-19 has shown the improvement of China’s global health technology and capability [13].

Due to the rapidly spreading of COVID-19, the WHO declared the COVID-19 outbreak a Public Health Emergency of International Concern on 30 January 2020 [14]. Worldwide, the COVID-19 pandemic has infected 9,843,073 cases and lead to 495,760 deaths by 10:00 CEST, 20 June 2020, according to the WHO [15]. Specifically, by the WHO region, Americas, Europe and Eastern Mediterranean had higher amount of confirmed cases (deaths) of COVID-19, 4,933,972 (241,931), 2,656,437 (196,541) and 1,024,222 (23,449), respectively [15]. 

### 2.2. Literature Review

Zhang and Shaw [1] investigate the content of coronavirus-related research published in the journals indexed in the Science Citation Index Expanded and Social Sciences Citation Index from 2000 to 2020. Furthermore, the textual analysis shows that coronavirus-related research mainly focuses on virology, immunology, epidemiology, and so forth. However, there are few studies that discuss the risk assessment. In addition, in Di Gennaro et al.’s [16] review paper on COVID-19, they focus on the literature about epidemiology, pathophysiology, diagnosis, management, and future perspective, but not the social and economic impacts of COVID-19. 

Regarding prior studies on investigating the social impacts of the COVID-19 outbreak, Ahmed et al.’s [17] questionnaire results demonstrate that dental practitioners who are working in the areas where face COVID-19 pandemic threats show a state of anxiety and fear, which suggests that COVID-19 outbreak has a negative effect on sentiment. Similar survey results show in Israeli dentists and dental hygienists [18]. In addition, Auerbach and Miller [19] highlight a severe issue regarding the shortage of mental health professionals due to the coronavirus pandemic. Li et al. [20] and Wang et al. [21] also illustrate that social risks lead to negative psychological consequences with increasing negative emotions. Besides, Chen et al. [22] document that the COVID-19 oriented risk positively affects the behaviors of hand-washing and mask-wearing based on the survey data from primary school students in Wuhan, China. Moreover, He et al. [23] approve the evidence that discrimination and social exclusion occurred after the outbreak of COVID-19.

Regarding prior studies on investigating the economic impacts of COVID-19 outbreak, first, for the cross-country studies, Ashraf [24] finds the growth in the number of country-level confirmed cases of COVID-19 has a negative effect on stock markets based on the 64 countries over the period 22 January to 17 April 2020. In addition, Engelhardt et al. [7] confirm that news attention of COVID-19 associate with the stock markets’ decline. Also, Zhang et al. [4] show that the COVID-19 pandemic leads to an increase in global financial market risks based on the cross-country evidence. Moreover, Liu et al. conduct an event study method and find that stock markets affected by COVID-19 fell quickly after the COVID-19 outbreak. Second, for the single country studies, based on the statistical figure from India, Singh and Neog [25] illustrate that the COVID-19 outbreak leads to an economic contraction in terms of macro-economy, tourism, transportation, stock market, human capital, and trade. Al-Awadhi et al. [3] use Chinese data and find that daily new confirmed COVID-19 cases and deaths negatively affect stock returns. Based on the U.S. daily data Sharif et al. [26] show that COVID-19 leads to oil price volatility shock, economic policy uncertainty, and stock market volatility. Using U.K. data from 2 January to 20 May 2020, Griffith et al. [5] show the impact of COVID-19 on share prices differs from industries. 

However, the relationship between the regional continued increasing COVID-19 cases and the firms’ market reaction remains unexplored. Focusing on the early period of the COVID-19 outbreak in China, this study attempts to fill the gap and investigate the negative effect of continued increasing provincial public health threats (driven by COVID-19) on the local firms’ market performance. 

### 2.3. Hypothesis Development

#### 2.3.1. Main Hypothesis

Continued increasing public health threats can negatively affect cumulative abnormal return for the following reasons. First, prior studies show that environmental uncertainty will negatively influence investor valuations and investor sentiment [6,27,28]. Also, prior studies show that individual psychology is related to stock price valuation [29,30,31]. Moreover, based on the Sina-Weibo (Chinese microblogging website) content analysis, Han et al. [32] show that public sentiments are sensitively affected by the epidemic and social events. In the case of the COVID-19 outbreak in China, the continued increase in the amount of new confirmed cases in one specific provincial region will enhance the uncertainty of the firms’ short-term and long-term performance in this area, which negatively influences investor valuations of local firms.

Second, prior research shows that the outbreak of the disease would increase the economic cost and shrink the profits in international markets [33,34,35]. In addition, economic conditions would affect the investors’ expectations of risks [36]. In the case of the COVID-19 outbreak in China, the continued increase in the amount of new confirmed cases in one specific provincial region will enhance the local economic cost and then enhance the investors’ risk assessment.

Third, several studies show that event risks (e.g., pollution events, hurricane disasters) will have a negative effect on firm valuation [37,38,39]. Moreover, Liu et al. [40] emphasize that significant events lead to abrupt changes in stock prices and volatility, and investors are more likely to hold less risky assets. In the case of the COVID-19 outbreak in China, the continued increase in the amount of new confirmed cases in one specific provincial region may increase the event risk and investors would be less likely to hold the financial assets from that area. 

Overall, firms’ market performance may be negatively affected by the regional continued increasing public health threats. Based on the above discussions, the first hypothesis is as follows (in an alternative form):

**Hypothesis** **1** **(H1).**  *Firms located in the provinces where face continued increase of public health threats are more likely to have a poor stock market performance.*

Notwithstanding the above arguments, there are a few reasons why firms’ market performance may not be negatively affected by COVID-19 situations. First, some investors might not be aware of the risks of the continued increase of COVID-19 cases. Even if investors have this awareness, they would still focus on the long-term performance of their investment portfolios. Second, the COVID-19 outbreak may bring opportunities to firms for generating more products to meet the increased demand currently and in the near future, which potentially leads to a positive effect on the performance. Third, investors might not focus on the daily based non-financial information from the National Health Commission of the People’s Republic of China, which leads to less value relevance for the COVID-19 disclosure. Taken together, whether the results consistent with H1 is an empirical question.

#### 2.3.2. Cross-Sectional Analyses

To support the theory and main hypothesis that the regional continued increasing public health threats influence the local firms’ market performance by enhancing the environmental uncertainty and investors’ risk assessment, we propose two sets of cross-sectional predictions that analyze the variation in the regional public health threats-oriented uncertainty and risk assessment.

One crucial channel underlying H1 is that continued increasing provincial public health threats can influence investor sentiment, thus enhance the risk assessment. Prior research suggests that information asymmetry has an effect on investor sentiment [41,42,43]. Specifically, Schmeling [42] finds that information asymmetry amplifies the negative effect of sentiment on future stock returns based on the cross-country evidence. In the case of regional information accessibility, we conjecture that if the investor could access more regional information, they will be less sentiment about increasing threats. We expect that stronger information accessibility will decrease the investors’ risk assessment by mitigating the information asymmetry. Consistent with this notion, Chakravarty et al. [44] apply the number of news reports as an inverse measure of information asymmetry and find that the number of news reports reduces the magnitude of the price discount. In addition, Bonsall et al. [45] find that wider media coverage, in terms of the news article number regarding earnings announcement, relates to the improvement in investor informedness during periods of higher market uncertainty. Base on the discussion, our first cross-sectional hypothesis is as follows (in an alternative form):

**Hypothesis** **2a** **(H2a).**  *The negative effect of continued increasing provincial public health threats on market reaction, as hypothesized in H1, is less pronounced when the provincial information accessibility is stronger.*

For the primary hypothesis, we assume that provincial continued increasing threats reduce the local firms’ stock market performance because such circumstances can enhance the uncertainty and investors’ risk assessment. However, if such circumstances occurred in a province where shows strong economic growth, then investors will have a lower level of risk assessment to constrain the investment. Prior studies documents that long-term equity premium is related to economic growth [46,47]. Moreover, Ludvigson [48] shows that economic activities relate to consumer confidence, and Chen [49] finds that a lack of consumer confidence leads to a higher likelihood of turning to a bearish stock market. Taken together, we suppose that stronger economic growth will decrease the investors’ risk assessment by enhancing the likelihood to have a positive outlook on the future market performance. Our second cross-sectional hypothesis is as follows (in an alternative form):

**Hypothesis** **2b** **(H2b).**  *The negative effect of continued increasing provincial public health threats on market reaction, as hypothesized in H1, is less pronounced when the provincial economic growth is stronger.*

## 3. Research Design

### 3.1. Sample Selection

To test our hypotheses, we use the Chinese setting from 10 January to 31 March 2020. The financial and executive data is collected from the CSMAR database; the COVID-19 data is collected from the National Health Commission of the People’s Republic of China; the stock price is collected from the RESSET database; and the province-level data is collected from the National Bureau of Statistics. Panel A in Table 1 shows the sample selection process. After dropping the sample with missing data, the final sample contains 178,805 firm-day observations.

Panel B of Table 1 shows the sample distribution by month. There are 237 firm-day observations for firms located in provinces face a continued increase in public health threats (*CIPHT* = 1) in January. The sample group of *CIPHT* = 1 increased dramatically during February, while there is a declining trend in the group of *CIPHT* = 1 during March. Panel C of Table 1 shows the sample distribution by industry. Firms from the manufacturing industry dominate the full sample, which is consistent with China’s industrial structure. The proportion of cross-industry distribution in sub-samples is similar to the full sample.

### 3.2. The Measure of Continued Increasing Public Health Threats

We attempt to evaluate public health threats using a continued increase of provincial COVID-19 cases to ascertain the investors’ reaction to the local increasing health threats. Specifically, we generate an indicator variable (*CIPHT*) to represent the regional increasing health threats. Here *CIPHT* equals one if there have been provincial new COVID-19 cases for at least six consecutive days including the current day and zero otherwise. The information on daily province-level new confirmed COVID-19 cases is collected from the National Health Commission of the People’s Republic of China. The COVID-19 disclosure news started from 11 January and contained data from 10 January. The daily based distribution of *CIPHT* = 1 sample is listed in Appendix B. 

### 3.3. The Measure of Market Reaction

We apply the cumulative abnormal return to represent the short-window market reaction to the continued increase of public health threats. Particularly, following the prior studies [38,50,51,52,53], we compute two measures of the firm’s cumulative abnormal return (*CAR*) with a three-day [−1, 1] window and a five-day [−2, 2] window based on the market model as follows:

*Firm Return* = *β_0_ + β_1_Market Return + ε* (1)

where *Firm Return* is the firm’s daily stock return, and *Market Return* is the daily stock market return. Similar to the prior studies [51,54], we estimate the value of the constant term (*β*_0_) and the systematic risk of the stock (*β*_1_) based on model (1) over the period from current day 200 to current day 60 ([−200, −60]) and day 0 is the date of the current day. Then we get the abnormal returns by calculating the residuals of model (1) with the estimated value of the constant term and systematic risk of the stock. Finally, we generate two types of cumulative abnormal returns around the three-day and five-day short windows (*CAR* [−1, 1] and *CAR* [−2, 2]). These two short-window abnormal return measures capture investors’ risk assessment of expected costs of the continued increasing public health threats.

### 3.4. Empirical Model

We describe the regression model for the main test of H1. The regression models for cross-sectional tests are described in Section 5. To test H1, we apply the multiple regression model as follows:

*CAR = β_0_ + β_1_CIPHT + β_2_PRO_CASE + β_3_SIZE + β_4_ROA + β_5_CURR + β_6_R&D + β_7_LOSS* *+ β_8_LEV + β_9_OPCF + β_10_TURN + β_11_CEO_AGE+ β_12_CEO_COM + β_13_CEO_TEN* *+ β_14_CEO_DUA + Week FE + Industry FE + Province FE + ε* (2)

where *CAR* refers to our two types of accumulative abnormal return (*CAR* [−1, 1] and *CAR* [−2, 2]), *CIPHT* is an indicator variable that equals one if there have been provincial new COVID-19 cases for at least six consecutive days including the current day and zero otherwise. Based on H1, we suppose a negative coefficient of *CIPHT*. 

Model (2) contains several determinants of accumulative abnormal return. Considering that provincial accumulated COVID-19 cases would affect the investors’ risk assessment, we add *PRO_CASE* into our model. *PRO_CASE* is the six-day mean value of the provincial ratio of the daily accumulated confirmed COVID-19 cases to the resident population. Moreover, along with prior studies, we control the firm attributes that will affect abnormal return [39,55,56]. *SIZE* is the natural logarithm of total assets; *ROA* is the return on assets; *CURR* is the current ratio; *R&D* is the ratio of R&D expenses to sales; *LOSS* is an indicator variable that equals one if the firm suffered a loss and zero otherwise; *LEV* is the leverage ratio of total liabilities to total assets; *OPCF* is the ratio of the firm’s operating cash flow to total assets; *TURN* is the asset turnover ratio. In addition, following prior studies [55,57,58,59], we add CEO attributes that will affect the market reaction. *CEO_AGE* is the age of the firm’s CEO; *CEO_COM* is the ratio of the firm’s CEO compensation to the net income; *CEO_TEN* is the tenure of the firm’s CEO that is defined as days of CEO’s tenure divided by 365; *CEO_DUA* is an indicator variable that equals one if the firm’s CEO holds a concurrent post in other work units and zero otherwise. Finally, we add week fixed effects, industry fixed effects, and province fixed effects. Appendix A presents detailed variable definitions. 

### 3.5. Descriptive Statistics

Panel A of Table 2 presents the descriptive statistics on all variables in the model (2) for the full sample. The median values of the *CAR* [−1, 1] and *CAR* [−2, 2] are −0.004 and −0.005, which suggests that during the COVID-19 outbreak period, more than half of the assessments of the firm performance are negative. The mean value of *CIPHT* is 0.360, which shows that there is 36.0 percent of the observations show the continued increasing public health threats. Panel B of Table 2 reports mean difference test between sub-samples (*CIPHT* = 0 vs. *CIPHT* = 1). The mean values of *CAR* [−1, 1] and *CAR* [−2, 2] are significantly lower for firm-days with continued increasing public health threats (−0.001 and −0.001) than for those without continued increasing public health threats (0.000 and 0.000). This result provides preliminary support on the negative relationship between continued increasing public health threats and abnormal return. Regarding the determinations of accumulative abnormal return, we find that firms facing continued increasing public health threats are located in the provinces where have more confirmed accumulated COVID-19 cases; have the larger size and better performance; have lower R&D ratio and leverage ratio; and hire CEOs with younger age, higher compensation, longer tenure, and a higher likelihood to hold a concurrent post.

Table 3 reports the correlation matrix of the variables in the main tests—Pearson correlations in the lower diagonal and Spearman correlations in the upper diagonal. For the Pearson correlation, the two measures of cumulative abnormal return (*CAR* [−1, 1] and *CAR* [−2, 2]) are significantly and negatively correlated with provincial continued increasing public health threats (*CIPHT*), which is consistent with H1 that increasing provincial threats positively affect the risk assessment of the investor. Given that the results in Table 3 are pairwise univariate correlations, we focus the primary analyses based on the multivariate tests in the next section. For multivariate tests, we calculate the VIF for each variable in the regression model (2) and find that all VIF values of variables, exclude the fixed effects, are less than 4. Thus, our multivariate analyses are not subject to a multicollinearity problem.

## 4. Main Analyses―Tests of H1

### 4.1. Full Sample Analyses

Table 4 presents the multiverse regression results on the association between continued increasing public health threats and accumulative abnormal return. The Columns (A) and (D) show the results of the full sample with the dependent variable *CAR*, test variable *CIPHT*, and fixed effects. The Columns (B) and (E) add nine variables that affect the cumulative abnormal return. Moreover, Columns (C) and (F) further add four CEO attributes that affect the cumulative abnormal return. We find that the coefficients on *CIPHT* in all columns are all negative and significant. These results indicate that the accumulative abnormal return is lower for firm-days with the continued increase in provincial public health threats, consistent with H1. Regarding the economic significance, the magnitude of the coefficients in Columns (C) and (F) suggest that the accumulative abnormal return (*CAR* [−1, 1] and *CAR* [−2, 2]) of a firm surrounded by a continued increasing public health threat is on average about 0.1 percent lower than that of a firm without continued regional increasing threats. 

### 4.2. Alternative Measures of Provincial Public Health Threats

Considering the potential measurement bias on test variables might drive the empirical results, we replace the *CIPHT* with alternative variables *CIPHT2* and *CIPHT3* and rerun the regression. Here, *CIPHT2* is an alternative variable for continued increasing public health threats that is measured as an indicator variable that equals one if there have been provincial new COVID-19 cases for at least seven consecutive days including the current day and zero otherwise; and *CIPHT3* is an alternative variable for continued increasing public health threats that is measured as an indicator variable that equals one if there have been provincial new COVID-19 cases for at least five consecutive days including the current day and zero otherwise. Table 5 shows the relation between alternative measures of continued increasing public health threats and accumulative abnormal return. Both alternative measures (i.e., *CIPHT2* and *CIPHT3*) are negatively and significantly related to *CAR* [−1, 1] and *CAR* [−2, 2], which reinforces our inference that continued increasing public health threats play an important role in increasing investors’ risk assessment.

### 4.3. Falsification Tests

In order to further strengthen the inferences, we conduct a falsification test. If the observed negative cumulative abnormal returns are indeed driven by the continued increase of public health threats, they are more likely to be concentrated around the days of the continued increase in the cases of COVID-19, not around days of a non-continued increase in the cases of COVID-19. Specifically, we use a different day as the pseudo-continued increase of public health threats day (*Pseudo_CIPHT*) and repeat the same analyses involving *CAR* [−1, 1] and *CAR* [−2, 2]. Here *Pseudo_CIPHT* is an indicator variable that equals one if there are no new cases for COVID-19 in a firm’s province on the current day, but new cases continued to occur in the last five days and zero otherwise. Table 6 reports the regressing results for this analysis. As reported in the table, the coefficient on *Pseudo_CIPHT* is insignificantly different from zero. It suggests that cumulative abnormal return does not negatively change for firms that do not face a continued increase of public health threats, providing further credence to the notion that the results reported in Table 4 are attributable to the continued increase of public health threats.

### 4.4. Endogeneity Concerns

To address the endogeneity concerns, we apply a two-stage least squares (2SLS) instrumental variable approach. In the first-stage regression, we regress the continued increase of public health threats on two instrument variables (*IMMIGRANT* and *EMIGRANT*). Here, *IMMIGRANT* is the six-day mean value of the ratio of the daily provincial immigrants to the national immigrants; and *EMIGRANT* is the six-day mean value of the ratio of the daily provincial emigrants to national emigrants. The daily mobility data is collected from the Baidu Migration website. Based on Jia et al. [10] and Kraemer et al.’s [9] findings, we argue that when the provincial immigrant (emigrant) rate is increased, the new COVID-19 cases are more (less) likely to be confirmed for the out-in (in-out) human mobility, enhancing (decreasing) the likelihood to face the continued increasing public health threats. We report the first-stage regression results in Column (A) of Table 7, where we regression *CIPHT* on all two instruments and the control variables added in the second-stage regression. We find that the coefficient of *EMIGRANT* is negative and significant, which consistent with our conjecture. Columns (B) and (C) of Table 7 report the second-stage regression results. We find that the coefficient of *Predicted_CIPHT*, estimated from the first-stage regression, is negatively and significantly associated with *CAR* [−1, 1] and *CAR* [−2, 2], respectively. Accordingly, the robust results based on the 2SLS approach mitigate endogeneity concerns and strengthen the main inference that continued increasing public health threats significantly influence market performance.

## 5. Cross-Sectional Analyses

### 5.1. Research Design

To test the H2a and H2b, we generate the regression model as follows:

*CAR = β_0_ + β_1_CIPHT + β_2_CIPHT × Conditioning_VAR + β_3_Conditioning_VAR* * + β_4_PRO_CASE + β_5_SIZE + β_6_ROA + β_7_CURR + β_8_R&D + β_9_LOSS + β_10_LEV * * + β_11_OPCF + β_12_TURN + β_13_CEO_AGE+ β_14_CEO_COM + β_15_CEO_TEN * *+ β_16_CEO_DUA + Week FE + Industry FE + Province FE + ε* (3)

where *Conditioning_VAR* is a conditioning variable that moderates the association between continued increasing public health threats and accumulative abnormal return. All other variables are above-mentioned. To test H2a and H2b, *Conditioning_VAR* is in terms of the provincial information accessibility and provincial economic growth, respectively. We explain the detail proxies in the following sections.

### 5.2. The Conditioning Effect of Provincial Information Accessibility―Test of H2a

Regrading H2a, we investigate whether the continued increasing public health threats in decreasing the accumulative abnormal return is weaker in firms that located in the provinces with stronger information accessibility. We suppose that stronger information accessibility will decrease the investors’ risk assessment by mitigating the information asymmetry. We apply three proxies (*High_WEB*, *High_MED*, and *High_MOB*) to represent stronger provincial information accessibility. Here, *High_WEB* is an indicator variable that equals one if the provincial ratio of the number of websites per 100 enterprises to resident population is higher than or equal to the upper quartile value and zero otherwise; *High_MED* is an indicator variable that equals one if the provincial TV coverage rate of population is higher than or equal to the upper quartile value and zero otherwise; and *High_MOB* is an indicator variable that equals one if the provincial ratio of flow accessed to mobile internet to resident population is higher than or equal to the upper quartile value and zero otherwise. For the H2a, we substitute *Conditioning_VAR* in Model (3) with *High_WEB*, *High_MED*, and *High_MOB*, respectively, and expect the coefficient of the interaction term is positive. Table 8 shows the regression results on H2a. We find that the interaction terms of *CIPHT × High_WEB*, *CIPHT × High_MED*, and *CIPHT × High_MOB* are positive and significant, which support the H2a that the negative effect of continued increasing provincial public health threats on market reaction is less pronounced when the provincial information accessibility is stronger (in terms of higher websites rate, higher media coverage, and higher mobile internet rate).

### 5.3. The Conditioning Effect of Provincial Economic Growth―Test of H2b

Regrading H2b, we investigate whether the continued increasing public health threats in decreasing the accumulative abnormal return is weaker in firms located in the provinces with stronger economic growth. We suppose that stronger economic growth will decrease the investors’ risk assessment by enhancing the likelihood to have a positive outlook on the future economy. We apply three proxies (*High_GRP*, *High_EMR*, and *High_URB*) to represent a stronger provincial economic growth. Here, *High_GRP* is an indicator variable that equals one if the provincial ratio of the gross regional product to resident population is higher than or equal to the upper quartile value and zero otherwise; *High_EMR* is an indicator variable that equals one if the provincial employment rate in the urban area is higher than or equal to the upper quartile value and zero otherwise; and *High_URB* is an indicator variable that equals one if the provincial ratio of urban population to resident population is higher than or equal to the upper quartile value and zero otherwise. For the H2b, we substitute *Conditioning_VAR* in Model (3) with *High_GRP*, *High_EMR*, and *High_URB*, respectively, and expect the coefficient of the interaction term is positive. Table 9 shows the regression results on H2b. We find that the interaction terms of *CIPHT × High_GRP*, *CIPHT × High_EMR*, and *CIPHT × High_URB* are positive and significant, which support the H2b that the negative effect of continued increasing provincial public health threats on market reaction is less pronounced when the provincial economic growth is stronger (in terms of higher gross regional product rate, higher employment rate, and higher urbanization).

## 6. Additional Analyses and Sensitivity Checks

### 6.1. Continued Decrease of Public Health Threats

To triangulate our results, we identify a situation that the firms located in a province where the threats of local public health are continually decreasing. The argument underlying H1 is that provincial increasing public health threats will lead to a lower level of accumulative abnormal return by enhancing the investors’ risk assessment. Presumably, if the province faces continued zero cases for newly confirmed COVID-19 that it does not have an adverse impact on decreasing public health threats to the local firms, investors will restrain the extent of risk assessment and investor trust can be expected to increase. Thus, we conjecture that continued decrease of provincial public health threats is positively related to the accumulative abnormal return. For testing this assumption, we substitute the *CIPHT* with *CDPHT* in Model (2) and run the regression. Here, *CDPHT* represents continued decreasing public health threats measured as an indicator variable that equals one if there have not been any provincial new COVID-19 cases for at least six consecutive days including the current day and zero otherwise. Table 10 presents the results of this assumption. We find a positive and significant coefficient on *CDPHT* in Columns (A) and (B), respectively, which consistent with our predictions.

### 6.2. The Effectiveness of Community Lockdown: Pre- versus Post-Lockdown Period

Considering the speedily spreading of COVID-19 in Wuhan province, Chinese governance decided to restrict human mobility by ordering a Wuhan lockdown since 23 January 2020. Moreover, China extends lockdown to more areas by implementing the “closed community management” measures. In February 2020, many provinces had selected the community lockdown mode. Prior research [9] finds that lockdown effectively mitigated the spread of COVID-19. We expect investors may notice the positive effects of lockdown and will restrain the risk assessment during the post-lockdown period. For testing this assumption, we substitute *Conditioning_VAR* in Model (3) with *POST_Lockdown*. Here *POST_Lockdown* is an indicator variable that equals one if the firm is in periods after implementing the "closed community management" measures and zero otherwise. The information of the lockdown periods by province is shown in the Appendix B. Table 11 shows the regression results, and we find that the coefficients of *CIPHT × POST_Lockdown* in both columns are positive and significant, which consistent with our assumption that community lockdown could mitigate the effect of continued increasing public health threats on accumulative abnormal return. 

### 6.3. The Impact of Firm-Level Characteristics

In an additional sensitivity test, we examine the firm heterogeneity in the effect of continued increasing public health threats. The first assumption is that firms with a lower level of local consumer demand or geographical concentration of local businesses are more likely to mitigate the business risk raised by local public health threat [60,61,62]. The second assumption is that firms with higher levels of operating cash flow are more likely to overcome the difficulty during the COVID-19 outbreak by improving the supply chain risk management [63] and investment diversification [64]. The third assumption is that compare to the firms with non-clean audit opinions, the firms with clean auditor opinions on their financial reports are more likely to gain investor trust by showing reliable financial information [65,66]. Following these arguments, we predict that firms with a higher level of foreign sales, operating cash flow, and with clean audit opinions are more likely to receive investor trust and have lower management risks. As such, continued increasing provincial public health threats is less useful for firms with such characteristics. To test our assumption, we substitute *Conditioning_VAR* in Model (3) with *High_FSALE*, *High_OPCF*, and *Clean_OPIN*, respectively. Here, *High_FSALE* is an indicator variable that equals one if the firm’s foreign sales are higher than or equal to the upper quartile value and zero otherwise; *High_OPCF* is an indicator variable that equals one if the ratio of the firm’s operating cash flow to total assets is higher than or equal to the upper quartile value and zero otherwise; and *Clean_OPIN* is an indicator variable that equals one if the firm received a clean audit opinion for its financial report and zero otherwise. 

Table 12 shows the regression results of the moderate effects of firm-level characteristics. We find that the coefficients of *CIPHT × High_FSALE*, *CIPHT × High_OPCF*, and *CIPHT × Clean_OPIN* are all positive and significant, which is consistent with our prediction that geographical diversification, cash flow efficiency, and reporting quality could mitigate the negative effect of continued increasing provincial public health threats on market reaction.

### 6.4. The Impact of Volatility of Provincial Increase in New COVID-19 Cases

As a final robustness check, we examine whether our results are influenced by the volatility of provincial increase in new confirmed COVID-19 cases because high volatility of the number changing in the new confirmed cases may affect the extent of investors’ risk assessment. To tackle this concern, we substitute *Conditioning_VAR* in Model (3) with *High_Volatility*. Here *High_Volatility* is an indicator variable that equals one if the six-day standard deviation of the new confirmed COVID-19 cases is higher than or equal to the upper quartile value and zero otherwise. Table 13 shows the regression results on the moderate effect of volatility of the new confirmed COVID-19 cases. We find that the coefficients of *CIPHT ×* *High_Volatility* are all statistically insignificant, suggesting that our results are not driven by the fluctuations in the number of new confirmed cases over time. 

## 7. Conclusions and Discussion

The issue of the economic outcomes of the COVID-19 outbreak has gained considerable attention in the world. However, how exactly the continued increasing provincial cases of COVID-19 affects the firms’ market performance is not entirely understood. In this paper, we examine whether and how the continued increase of public health threats negatively affects the cumulative abnormal return.

Using the 178,805 firm-day observations from Chinese listed firms from 10 January to 31 March 2020, we find that the accumulative abnormal return is significantly lower among firms located in the provinces where face the continued increase of new confirmed COVID-19 cases. The relations remain constant after several robustness tests. These findings suggest that investors concern about the potential risk when firms are located in the provinces with higher threats to public health. In addition, we find that the relation between increasing provincial public health threat and firms’ abnormal return is affected by the regional information accessibility and economic growth. 

Moreover, we conduct several additional tests to ensure the robustness of our results. First, we find that the continued decrease of provincial public health threats is positively related to the accumulative abnormal return. Second, we find that the community lockdown has a negative moderating effect on the negative relationship between continued increasing public health threats and accumulative abnormal return. Third, we find that the negative effect of increasing threats is weaker for firms with a higher level of geographical diversification, operating cash flow, and with a clean audit opinion. Fourth, we find that our results are not driven by the fluctuations in the number of new confirmed COVID-19 cases.

This study contributes to the literature in many aspects. First, we add to the growing literature on the role that public health threats play in market reactions. Prior studies contend that the firms in heavy-polluting industries have a negative market reaction to the passage of the Environmental Protection Tax Law [39]; negative cumulative abnormal returns occur during short windows around pollution events [38]; and insurance firms have higher abnormal returns around the event window in most of the hurricane hazards [37]. These findings suggest that the increase in public health risks affects the markets. We add to this body of research by showing that the continued increase of COVID-19 cases at the provincial level will negatively affect the firm’s market performance by enhancing the investors’ risk assessment.

Second, we also contribute to the literature on risk assessment on COVID-19 and provide evidence that continued increasing regional public health threats are an essential determinant of local firms’ share prices. This study answers Zhang and Shaw’s [1] call for multi-disciplinary research incorporating public health and socioeconomics. Our findings shed light on this observation and suggest that the provincial-level threats of public health play a substantial role in determining local firms’ market performance. Moreover, compared to the prior COVID-19 related studies based on the evidence from China’s market [3], this study uses a much larger sample to test the effect of continued increasing public health threats, which enhance the statistical power. Considering the limitations of the cross-country setting [2,4,7,24], this study supplements prior studies by focusing on one single market. 

Third, we add the literature on the moderate effects of macro factors on public health threats. This study shows that provincial-based continued increased cases of COVID-19 affect investors’ expectations of the local firms’ exposure to increased risk of public health. Moreover, our cross-sectional analyses show that investors’ risk assessment could be driven by the provincial level of information asymmetry and economic outlook.

Fourth, we extend the literature on information disclosure and show the usefulness of the timely disclosure on disease information from the governmental institution. It will be helpful to the investor for facilitating decision making. This study provides evidence that the continued increase of provincial new confirmed COVID-19 cases is an essential signal for investors.

This study has several implications for interested parties. Investors should realize the usefulness of the non-financial information and pay more attention to the detailed information related to public health threats. Companies need to enhance the level of geographical diversification, operating cash flow, and reporting quality for mitigating the negative effect of local public health threats on their market performance. For the government and policymakers, they should understand the important moderate effects of the local environment on the negative effect of public health threats and take the balance of the cross-provinces development from the perspectives of the information technology and economics. 

Our study has several limitations that could be addressed by future studies. First, we add several cross-sectional tests, the instrumental variables approach, and many control variables to mitigate the endogeneity concerns. However, it is difficult to rule out all confounding factors using archival data. Second, the abnormal return measures are inherently limited and may not entirely represent abnormal returns. Third, given that the institutional characteristics of China are different from other countries, the negative effect of continued increasing public health threats may not exist in other settings. Fourth, considering the difficulty of the measurement for the regional public health threats, information accessibility, and economic growth (e.g., we fail to measure the quality of news information), we merely provide some testable proxies to represent these concepts and show that they relate to the investors’ risk assessment on continued public health threats.

## Figures and Tables

**Table 1:** Sample restriction and distribution.

**Panel A: Sample Restriction**			
		**Firms**	**Firm-Days**
**Description**		**Obs.**	**Obs.**
Observations from 10 January to 31 March 2020 with CSMAR and RESSET data		3635	187,030
Less: financial companies		−108	−5522
Less: missing data for measuring variables		−52	−2703
Final sample		3475	178,805
**Panel B: Distribution by Month**			
	**Firm-Days**	***CIPHT* = 0**	***CIPHT* = 1**
**Month**	**Obs.**	**Obs.**	**Obs.**
January	34,147	33,910	237
February	68,645	21,675	46,970
March	76,013	58,875	17,138
Total	178,805	114,460	64,345
**Panel C: Distribution by Industry**			
	**Firm-Days**	***CIPHT* = 0**	***CIPHT* = 1**
**Industry**	**Obs.**	**Obs.**	**Obs.**
Accommodation and food	468	300	168
Comprehensive	1092	706	386
Construction	4775	2787	1988
Culture, sports, and entertainment	2953	1808	1145
Education	416	236	180
Farming, forestry, animal husbandry, and fishery	2027	1464	563
Health and social work	572	353	219
Information transmission, software, and information technology services	14,413	8582	5831
Leasing and business services	2688	1514	1174
Manufacturing	115,676	74,983	40,693
Mining	3578	2516	1062
Production and supply of electricity, heat, gas, and water	5344	3573	1771
Real estate	5903	3559	2344
Resident services, repair, and other services	52	27	25
Scientific research and technical services	2798	1747	1051
Transportation, warehousing and postal services	5248	3405	1843
Water conservancy, environment, and public institution management	2648	1769	879
Wholesale and retail trade	8154	5131	3023
Total	178,805	114,460	64,345

*Note*: This table presents the sample restriction and distribution by month/industry. Panel A presents the sample restriction for the period from 10 January to 31 March 2020. Panel B presents the descriptive statistics of the sample over three months. Panel C presents an industry breakdown of the sample based on the China Securities Regulatory Commission industry classification in 2012. See Appendix A for definitions and measurements of the variables.

**Table 2:** Descriptive statistics.

**Panel A: Descriptive Statistics**					
	**Full sample (*N* = 178,805)**				
**Variable**	**Mean**	**Std. Dev.**	**Q1**	**Median**	**Q3**
*CAR* [−1, 1]	0.000	0.046	−0.025	−0.004	0.020
*CAR* [−2, 2]	0.000	0.061	−0.034	−0.005	0.027
*CIPHT*	0.360	0.480	0.000	0.000	1.000
*PRO_CASE*	0.265	1.327	0.034	0.079	0.147
*SIZE*	22.280	1.359	21.343	22.096	23.018
*ROA*	0.013	0.652	0.008	0.025	0.053
*CURR*	2.480	3.159	1.152	1.641	2.666
*R&D*	0.249	12.999	0.000	0.000	0.000
*LOSS*	0.076	0.266	0.000	0.000	0.000
*LEV*	0.439	0.571	0.265	0.414	0.571
*OPCF*	0.035	0.108	0.002	0.026	0.064
*TURN*	0.463	0.886	0.156	0.341	0.564
*CEO_AGE*	52.191	5.851	49.000	52.500	56.000
*CEO_COM*	0.025	0.405	0.002	0.006	0.014
*CEO_TEN*	4.774	3.498	2.148	4.156	6.405
*CEO_DUA*	0.680	0.395	0.500	1.000	1.000
**Panel B: Mean Difference Test**					
	***CIPHT* = 0 vs. *CIPHT* = 1 (Mean)**				
**Variable**	***CIPHT* = 0**	***CIPHT* = 1**	**Diff.**	**t-statistic**	
*CAR* [−1, 1]	0.000	−0.001	0.001	3.26	***
*CAR* [−2, 2]	0.000	−0.001	0.001	3.99	***
*CIPHT*	0.000	1.000	−1.000		
*PRO_CASE*	0.158	0.455	−0.297	−45.65	***
*SIZE*	22.274	22.292	−0.018	−2.69	***
*ROA*	0.013	0.013	0.001	0.22	
*CURR*	2.483	2.476	0.007	0.43	
*R&D*	0.318	0.125	0.193	3.01	***
*LOSS*	0.078	0.074	0.004	3.43	***
*LEV*	0.441	0.436	0.005	1.82	*
*OPCF*	0.035	0.035	0.000	−0.25	
*TURN*	0.463	0.464	−0.002	−0.38	
*CEO_AGE*	52.243	52.097	0.146	5.07	***
*CEO_COM*	0.022	0.029	−0.006	−3.13	***
*CEO_TEN*	4.750	4.815	−0.065	−3.74	***
*CEO_DUA*	0.678	0.683	−0.004	−2.14	**

*Note:* *, **, *** indicate significance at the 0.10, 0.05, and 0.01 levels, respectively. See Appendix A for definitions and measurements of the variables.

**Table 3:** Correlation matrix.

**Panel A: Correlation Variables *CAR* [** **−1, 1] to *R&D***									
		**1**	**2**	**3**	**4**	**5**	**6**	**7**	**8**
1	*CAR* [−1, 1]		**0.761**	0.000	**−0.010**	**0.022**	**−0.016**	**−0.017**	**−0.007**
2	*CAR* [−2, 2]	**0.792**		0.001	**−0.006**	**0.024**	**−0.019**	**−0.021**	**−0.008**
3	*CIPHT*	**−0.008**	**−0.009**		**0.267**	0.002	**0.011**	**0.012**	**0.017**
4	*PRO_CASE*	**0.010**	**0.013**	**0.107**		**0.008**	**0.025**	**0.032**	**0.022**
5	*SIZE*	0.004	**0.005**	**0.006**	**−0.008**		**−0.323**	**−0.440**	**0.174**
6	*ROA*	−0.002	−0.003	−0.001	0.003	**0.065**		**0.398**	**−0.117**
7	*CURR*	**−0.011**	**−0.015**	−0.001	**−0.005**	**−0.271**	**0.025**		**−0.063**
8	*R&D*	**0.006**	**0.007**	**−0.007**	−0.003	**0.014**	0.003	**−0.006**	
9	*LOSS*	−0.001	−0.002	**−0.008**	**0.012**	**−0.065**	**−0.144**	**−0.009**	−0.004
10	*LEV*	0.004	**0.005**	−0.004	−0.003	**0.103**	**−0.275**	**−0.208**	0.000
11	*OPCF*	**−0.012**	**−0.016**	0.001	**−0.031**	**−0.090**	**0.075**	**0.066**	**−0.006**
12	*TURN*	0.000	0.000	0.001	**−0.015**	**−0.182**	**−0.046**	**−0.014**	**−0.010**
13	*CEO_AGE*	0.002	0.003	**−0.012**	**−0.019**	**0.107**	**0.025**	−0.003	**0.013**
14	*CEO_COM*	0.001	0.001	**0.007**	**0.007**	**0.024**	0.000	**−0.013**	0.000
15	*CEO_TEN*	**0.006**	**0.008**	**0.009**	−0.004	**0.062**	**−0.016**	**−0.015**	**−0.015**
16	*CEO_DUA*	**−0.005**	**−0.006**	**0.005**	**−0.038**	**0.030**	**0.035**	**0.024**	**−0.007**
**Panel B: Correlation Variables *LOSS* to *CEO_DUA***									
		**9**	**10**	**12**	**11**	**13**	**14**	**15**	**16**
1	*CAR* [−1, 1]	**−0.005**	**0.019**	**−0.017**	**−0.009**	**0.008**	−0.002	**0.007**	**−0.006**
2	*CAR* [−2, 2]	**−0.006**	**0.024**	**−0.023**	**−0.011**	**0.009**	0.001	**0.009**	**−0.007**
3	*CIPHT*	**−0.008**	0.000	0.001	0.001	**−0.009**	**0.016**	**0.009**	**0.006**
4	*PRO_CASE*	**−0.006**	**−0.014**	**0.021**	0.004	**−0.009**	**0.013**	**0.009**	**0.023**
5	*SIZE*	**−0.052**	**0.465**	**−0.159**	**−0.325**	**0.089**	**−0.274**	**0.042**	0.002
6	*ROA*	**−0.460**	**−0.426**	**0.338**	**0.482**	**0.056**	**−0.125**	**0.021**	**0.081**
7	*CURR*	**−0.065**	**−0.815**	**0.183**	**0.113**	**0.036**	**0.077**	**0.047**	**0.040**
8	*R&D*	0.003	**0.056**	**−0.109**	**−0.165**	**0.060**	**0.018**	**0.083**	**0.022**
9	*LOSS*		**0.071**	**−0.069**	**−0.038**	**−0.066**	**−0.460**	**−0.057**	**−0.047**
10	*LEV*	**0.080**		**−0.285**	**−0.126**	**−0.045**	**−0.059**	**−0.063**	**−0.045**
11	*OPCF*	**−0.022**	**−0.132**		**0.206**	**0.026**	**−0.077**	**−0.014**	**0.042**
12	*TURN*	**0.024**	**0.116**	**0.142**		**−0.006**	**−0.175**	**−0.046**	**0.032**
13	*CEO_AGE*	**−0.065**	**−0.066**	**−0.012**	**−0.046**		0.004	**0.269**	**0.066**
14	*CEO_COM*	**−0.038**	**0.009**	**−0.010**	**−0.018**	**0.007**		**0.075**	−0.002
15	*CEO_TEN*	**−0.049**	**−0.037**	**−0.048**	**−0.062**	**0.283**	−0.003		**0.116**
16	*CEO_DUA*	**−0.043**	**−0.026**	**0.008**	**−0.015**	**0.067**	**0.012**	**0.101**	

*Note*: Correlations are based on 178,805 firm-day observations. Pearson (Spearman) correlations in the lower (upper) diagonal. Correlation coefficients in bold are statistically significant at the 0.10 level or better. See Appendix A for definitions and measurements of the variables.

**Table 4:** Continued increasing public health threats and accumulative abnormal return.

	*CAR* [−1, 1]	*CAR* [−1, 1]	*CAR* [−1, 1]	*CAR* [−2, 2]	*CAR* [−2, 2]	*CAR* [−2, 2]
	(A)	(B)	(C)	(D)	(E)	(F)
*CIPHT*	−0.000 *	−0.001 **	−0.001 **	−0.001 **	−0.001 ***	−0.001 ***
	(−1.716)	(−2.375)	(−2.372)	(−2.033)	(−2.868)	(−2.865)
*PRO_CASE*		0.000 ***	0.000 ***		0.001 ***	0.001 ***
		(3.066)	(3.065)		(3.885)	(3.882)
*SIZE*		−0.000	−0.000		−0.000	−0.000
		(−0.386)	(−0.587)		(−0.728)	(−0.978)
*ROA*		−0.000	−0.000		−0.000	−0.000
		(−1.118)	(−0.986)		(−1.365)	(−1.196)
*CURR*		−0.000 ***	−0.000 ***		−0.000 ***	−0.000 ***
		(−3.731)	(−3.670)		(−4.843)	(−4.764)
*R&D*		0.000 **	0.000 **		0.000 ***	0.000 ***
		(2.473)	(2.519)		(3.062)	(3.122)
*LOSS*		−0.001	−0.001		−0.001 **	−0.001 *
		(−1.622)	(−1.549)		(−1.993)	(−1.902)
*LEV*		−0.000	−0.000		−0.000	−0.000
		(−1.133)	(−1.010)		(−1.284)	(−1.132)
*OPCF*		−0.004 ***	−0.004 ***		−0.008 ***	−0.008 ***
		(−4.259)	(−4.128)		(−5.722)	(−5.553)
*TURN*		0.000	0.000		0.000	0.000
		(0.757)	(0.865)		(0.992)	(1.130)
*CEO_AGE*			0.000			0.000
			(0.135)			(0.079)
*CEO_COM*			0.000			0.000
			(0.239)			(0.300)
*CEO_TEN*			0.000 ***			0.000 ***
			(2.742)			(3.556)
*CEO_DUA*			−0.000			−0.001
			(−1.253)			(−1.575)
Constant	−0.001	0.002	0.002	−0.002	0.003	0.004
	(−0.336)	(0.404)	(0.515)	(−0.484)	(0.561)	(0.722)
Week FE	Yes	Yes	Yes	Yes	Yes	Yes
Industry FE	Yes	Yes	Yes	Yes	Yes	Yes
Province FE	Yes	Yes	Yes	Yes	Yes	Yes
Adjusted R^2^	0.003	0.003	0.003	0.004	0.005	0.005
Observations	178,805	178,805	178,805	178,805	178,805	178,805

*Note*: *, **, *** indicate significance at the 0.10, 0.05, and 0.01 levels, respectively. See Appendix A for definitions and measurements of the variables.

**Table 5:** Alternative measures of continued increasing public health threats and accumulative abnormal return.

	*CAR* [−1, 1]	*CAR* [−1, 1]	*CAR* [−2, 2]	*CAR* [−2, 2]
	(A)	(B)	(C)	(D)
*CIPHT2*	−0.001 **		−0.001 **	
	(−2.271)		(−2.464)	
*CIPHT3*		−0.001 **		−0.002 ***
		(−2.324)		(−3.079)
*PRO_CASE*	0.000 ***	0.000 ***	0.001 ***	0.001 ***
	(3.107)	(3.014)	(3.911)	(3.831)
*SIZE*	−0.000	−0.000	−0.000	−0.000
	(−0.587)	(−0.587)	(−0.978)	(−0.978)
*ROA*	−0.000	−0.000	−0.000	−0.000
	(−0.986)	(−0.986)	(−1.196)	(−1.196)
*CURR*	−0.000 ***	−0.000 ***	−0.000 ***	−0.000 ***
	(−3.671)	(−3.670)	(−4.765)	(−4.763)
*R&D*	0.000 **	0.000 **	0.000 ***	0.000 ***
	(2.519)	(2.519)	(3.122)	(3.122)
*LOSS*	−0.001	−0.001	−0.001 *	−0.001 *
	(−1.549)	(−1.549)	(−1.902)	(−1.902)
*LEV*	−0.000	−0.000	−0.000	−0.000
	(−1.010)	(−1.010)	(−1.132)	(−1.131)
*OPCF*	−0.004 ***	−0.004 ***	−0.008 ***	−0.008 ***
	(−4.128)	(−4.127)	(−5.553)	(−5.553)
*TURN*	0.000	0.000	0.000	0.000
	(0.865)	(0.865)	(1.130)	(1.130)
*CEO_AGE*	0.000	0.000	0.000	0.000
	(0.135)	(0.135)	(0.079)	(0.079)
*CEO_COM*	0.000	0.000	0.000	0.000
	(0.239)	(0.239)	(0.300)	(0.300)
*CEO_TEN*	0.000 ***	0.000 ***	0.000 ***	0.000 ***
	(2.742)	(2.742)	(3.557)	(3.556)
*CEO_DUA*	−0.000	−0.000	−0.001	−0.001
	(−1.253)	(−1.253)	(−1.575)	(−1.575)
Constant	0.002	0.002	0.004	0.004
	(0.520)	(0.516)	(0.737)	(0.713)
Week FE	Yes	Yes	Yes	Yes
Industry FE	Yes	Yes	Yes	Yes
Province FE	Yes	Yes	Yes	Yes
Adjusted R^2^	0.003	0.003	0.005	0.005
Observations	178,805	178,805	178,805	178,805

*Note*: *, **, *** indicate significance at the 0.10, 0.05, and 0.01 levels, respectively. See Appendix A for definitions and measurements of the variables.

**Table 6:** Falsification tests.

	*CAR* [−1, 1]	*CAR* [−2, 2]
	(A)	(B)
*Pseudo_CIPHT*	0.000	0.000
	(0.358)	(0.225)
*PRO_CASE*	0.000 ***	0.001 ***
	(2.930)	(3.721)
*SIZE*	−0.000	−0.000
	(−0.587)	(−0.978)
*ROA*	−0.000	−0.000
	(−0.986)	(−1.196)
*CURR*	−0.000 ***	−0.000 ***
	(−3.673)	(−4.767)
*R&D*	0.000 **	0.000 ***
	(2.519)	(3.122)
*LOSS*	−0.001	−0.001 *
	(−1.548)	(−1.901)
*LEV*	−0.000	−0.000
	(−1.011)	(−1.132)
*OPCF*	−0.004 ***	−0.008 ***
	(−4.124)	(−5.548)
*TURN*	0.000	0.000
	(0.869)	(1.135)
*CEO_AGE*	0.000	0.000
	(0.134)	(0.078)
*CEO_COM*	0.000	0.000
	(0.240)	(0.301)
*CEO_TEN*	0.000 ***	0.000 ***
	(2.744)	(3.559)
*CEO_DUA*	−0.000	−0.001
	(−1.254)	(−1.576)
Constant	0.002	0.004
	(0.597)	(0.820)
Week FE	Yes	Yes
Industry FE	Yes	Yes
Province FE	Yes	Yes
Adjusted R^2^	0.003	0.005
Observations	178,805	178,805

*Note*: *, **, *** indicate significance at the 0.10, 0.05, and 0.01 levels, respectively. See Appendix A for definitions and measurements of the variables.

**Table 7:** Continued increasing public health threats and accumulative abnormal return based on the instrumental variables (2SLS) approach.

	*CIPHT*	*CAR* [−1, 1]	*CAR* [−2, 2]
	(A)	(B)	(C)
*Predicted_CIPHT*		−0.001 *	−0.003 ***
		(−1.844)	(−3.236)
*PRO_CASE*	−4.958 ***	0.001 ***	0.001 ***
	(−19.035)	(3.648)	(4.604)
*SIZE*	0.000	−0.000	−0.000 *
	(0.082)	(−1.374)	(−1.695)
*ROA*	−0.000	−0.000	−0.000
	(−0.040)	(−1.194)	(−1.456)
*CURR*	0.001	−0.000 ***	−0.000 ***
	(0.658)	(−4.119)	(−5.284)
*R&D*	0.000	0.000 **	0.000 ***
	(0.012)	(2.487)	(3.312)
*LOSS*	−0.001	−0.000	−0.001
	(−0.044)	(−0.924)	(−1.168)
*LEV*	0.001	−0.000	−0.000
	(0.047)	(−0.433)	(−0.562)
*OPCF*	−0.036	−0.005 ***	−0.009 ***
	(−0.661)	(−4.826)	(−6.114)
*TURN*	−0.004	0.000	0.000
	(−0.538)	(0.938)	(1.194)
*CEO_AGE*	0.000	−0.000	−0.000
	(0.160)	(−0.346)	(−0.600)
*CEO_COM*	−0.000	−0.000	0.000
	(−0.036)	(−0.203)	(0.007)
*CEO_TEN*	−0.001	0.000 ***	0.000 ***
	(−0.297)	(2.932)	(3.629)
*CEO_DUA*	0.001	−0.001 *	−0.001 **
	(0.060)	(−1.767)	(−2.420)
*IMMIGRANT*	−0.306 ***		
	(−49.316)		
*EMIGRANT*	−0.058 ***		
	(−5.352)		
Constant	3.869 ***	−0.005	−0.007
	(23.663)	(−0.721)	(−0.740)
Week FE	Yes	Yes	Yes
Industry FE	Yes	Yes	Yes
Province FE	Yes	Yes	Yes
Pseudo R^2^/Adjusted R^2^	0.744	0.003	0.005
Observations	160,744	160,744	160,744

*Note*: *, **, *** indicate significance at the 0.10, 0.05, and 0.01 levels, respectively. See Appendix A for definitions and measurements of the variables.

**Table 8:** Continued increasing public health threats and accumulative abnormal return conditioning on provincial information accessibility.

	*CAR* [−1, 1]	*CAR* [−1, 1]	*CAR* [−1, 1]	*CAR* [−2, 2]	*CAR* [−2, 2]	*CAR* [−2, 2]
	(A)	(B)	(C)	(D)	(E)	(F)
*CIPHT*	−0.002 ***	−0.002 ***	−0.002 ***	−0.002 ***	−0.004 ***	−0.003 ***
	(−3.570)	(−5.012)	(−4.678)	(−4.423)	(−6.268)	(−5.810)
*CIPHT × High_WEB*	0.002 ***			0.003 ***		
	(3.461)			(4.455)		
*High_WEB*	−0.002			−0.003		
	(−0.896)			(−1.024)		
*CIPHT × High_MED*		0.003 ***			0.004 ***	
		(5.500)			(7.032)	
*High_MED*		−0.002			−0.002	
		(−0.732)			(−0.908)	
*CIPHT × High_MOB*			0.003 ***			0.004 ***
			(5.163)			(6.550)
*High_MOB*			−0.001			−0.001
			(−0.363)			(−0.428)
*PRO_CASE*	0.000 ***	0.001 ***	0.001 ***	0.001 ***	0.001 ***	0.001 ***
	(3.109)	(3.247)	(3.249)	(3.940)	(4.116)	(4.116)
*SIZE*	−0.000	−0.000	−0.000	−0.000	−0.000	−0.000
	(−0.588)	(−0.586)	(−0.588)	(−0.979)	(−0.977)	(−0.979)
*ROA*	−0.000	−0.000	−0.000	−0.000	−0.000	−0.000
	(−0.985)	(−0.986)	(−0.986)	(−1.195)	(−1.196)	(−1.196)
*CURR*	−0.000 ***	−0.000 ***	−0.000 ***	−0.000 ***	−0.000 ***	−0.000 ***
	(−3.670)	(−3.661)	(−3.664)	(−4.763)	(−4.753)	(−4.757)
*R&D*	0.000 **	0.000 **	0.000 **	0.000 ***	0.000 ***	0.000 ***
	(2.519)	(2.519)	(2.519)	(3.122)	(3.122)	(3.122)
*LOSS*	−0.001	−0.001	−0.001	−0.001 *	−0.001 *	−0.001 *
	(−1.548)	(−1.549)	(−1.548)	(−1.900)	(−1.902)	(−1.901)
*LEV*	−0.000	−0.000	−0.000	−0.000	−0.000	−0.000
	(−1.009)	(−1.009)	(−1.010)	(−1.130)	(−1.129)	(−1.132)
*OPCF*	−0.004 ***	−0.004 ***	−0.004 ***	−0.008 ***	−0.008 ***	−0.008 ***
	(−4.122)	(−4.125)	(−4.141)	(−5.546)	(−5.550)	(−5.571)
*TURN*	0.000	0.000	0.000	0.000	0.000	0.000
	(0.871)	(0.872)	(0.861)	(1.138)	(1.140)	(1.125)
*CEO_AGE*	0.000	0.000	0.000	0.000	0.000	0.000
	(0.134)	(0.134)	(0.135)	(0.078)	(0.077)	(0.079)
*CEO_COM*	0.000	0.000	0.000	0.000	0.000	0.000
	(0.239)	(0.239)	(0.239)	(0.301)	(0.301)	(0.300)
*CEO_TEN*	0.000 ***	0.000 ***	0.000 ***	0.000 ***	0.000 ***	0.000 ***
	(2.742)	(2.743)	(2.740)	(3.557)	(3.558)	(3.554)
*CEO_DUA*	−0.000	−0.000	−0.000	−0.001	−0.001	−0.001
	(−1.254)	(−1.250)	(−1.250)	(−1.577)	(−1.572)	(−1.572)
Constant	0.003	0.002	0.003	0.005	0.004	0.005
	(0.762)	(0.530)	(0.829)	(0.972)	(0.741)	(1.103)
Week FE	Yes	Yes	Yes	Yes	Yes	Yes
Industry FE	Yes	Yes	Yes	Yes	Yes	Yes
Province FE	Yes	Yes	Yes	Yes	Yes	Yes
Adjusted R^2^	0.003	0.003	0.003	0.005	0.005	0.005
Observations	178,805	178,805	178,805	178,805	178,805	178,805

*Note*: *, **, *** indicate significance at the 0.10, 0.05, and 0.01 levels, respectively. See Appendix A for definitions and measurements of the variables.

**Table 9:** Continued increasing public health threats and accumulative abnormal return conditioning on provincial economic growth.

	*CAR* [−1, 1]	*CAR* [−1, 1]	*CAR* [−1, 1]	*CAR* [−2, 2]	*CAR* [−2, 2]	*CAR* [−2, 2]
	(A)	(B)	(C)	(D)	(E)	(F)
*CIPHT*	−0.001 ***	−0.001 ***	−0.002 ***	−0.002 ***	−0.002 ***	−0.003 ***
	(−3.459)	(−3.281)	(−3.546)	(−4.263)	(−4.103)	(−4.539)
*CIPHT × High_GRP*	0.002 ***			0.003 ***		
	(3.267)			(4.164)		
*High_GRP*	−0.000			0.000		
	(−0.074)			(0.132)		
*CIPHT × High_EMR*		0.001 ***			0.002 ***	
		(2.679)			(3.569)	
*High_EMR*		0.011 ***			0.018 ***	
		(4.437)			(5.556)	
*CIPHT × High_URB*			0.001 ***			0.003 ***
			(2.925)			(4.025)
*High_URB*			0.003			0.005 *
			(1.326)			(1.693)
*PRO_CASE*	0.000 ***	0.000 ***	0.000 ***	0.001 ***	0.001 ***	0.001 ***
	(3.109)	(3.146)	(3.182)	(3.939)	(3.990)	(4.044)
*SIZE*	−0.000	−0.000	−0.000	−0.000	−0.000	−0.000
	(−0.588)	(−0.587)	(−0.587)	(−0.979)	(−0.978)	(−0.978)
*ROA*	−0.000	−0.000	−0.000	−0.000	−0.000	−0.000
	(−0.985)	(−0.986)	(−0.986)	(−1.195)	(−1.196)	(−1.196)
*CURR*	−0.000 ***	−0.000 ***	−0.000 ***	−0.000 ***	−0.000 ***	−0.000 ***
	(−3.669)	(−3.667)	(−3.664)	(−4.763)	(−4.760)	(−4.756)
*R&D*	0.000 **	0.000 **	0.000 **	0.000 ***	0.000 ***	0.000 ***
	(2.519)	(2.519)	(2.519)	(3.122)	(3.122)	(3.122)
*LOSS*	−0.001	−0.001	−0.001	−0.001 *	−0.001 *	−0.001 *
	(−1.547)	(−1.548)	(−1.549)	(−1.900)	(−1.901)	(−1.902)
*LEV*	−0.000	−0.000	−0.000	−0.000	−0.000	−0.000
	(−1.009)	(−1.010)	(−1.009)	(−1.130)	(−1.132)	(−1.130)
*OPCF*	−0.004 ***	−0.004 ***	−0.004 ***	−0.008 ***	−0.008 ***	−0.008 ***
	(−4.121)	(−4.134)	(−4.126)	(−5.545)	(−5.561)	(−5.551)
*TURN*	0.000	0.000	0.000	0.000	0.000	0.000
	(0.871)	(0.863)	(0.869)	(1.138)	(1.128)	(1.136)
*CEO_AGE*	0.000	0.000	0.000	0.000	0.000	0.000
	(0.135)	(0.135)	(0.135)	(0.078)	(0.078)	(0.078)
*CEO_COM*	0.000	0.000	0.000	0.000	0.000	0.000
	(0.239)	(0.239)	(0.239)	(0.301)	(0.300)	(0.300)
*CEO_TEN*	0.000 ***	0.000 ***	0.000 ***	0.000 ***	0.000 ***	0.000 ***
	(2.742)	(2.742)	(2.742)	(3.557)	(3.556)	(3.557)
*CEO_DUA*	−0.000	−0.000	−0.000	−0.001	−0.001	−0.001
	(−1.253)	(−1.252)	(−1.251)	(−1.576)	(−1.574)	(−1.573)
Constant	0.003	0.002	0.002	0.005	0.004	0.004
	(0.761)	(0.522)	(0.521)	(0.972)	(0.731)	(0.730)
Week FE	Yes	Yes	Yes	Yes	Yes	Yes
Industry FE	Yes	Yes	Yes	Yes	Yes	Yes
Province FE	Yes	Yes	Yes	Yes	Yes	Yes
Adjusted R^2^	0.003	0.003	0.003	0.005	0.005	0.005
Observations	178,805	178,805	178,805	178,805	178,805	178,805

*Note*: *, **, *** indicate significance at the 0.10, 0.05, and 0.01 levels, respectively. See Appendix A for definitions and measurements of the variables.

**Table 10:** Continued decreasing public health threats and accumulative abnormal return.

	*CAR* [−1, 1]	*CAR* [−2, 2]
	(A)	(B)
*CDPHT*	0.003 ***	0.004 ***
	(6.511)	(7.598)
*PRO_CASE*	0.001 ***	0.001 ***
	(3.443)	(4.318)
*SIZE*	−0.000	−0.000
	(−0.589)	(−0.980)
*ROA*	−0.000	−0.000
	(−0.987)	(−1.197)
*CURR*	−0.000 ***	−0.000 ***
	(−3.661)	(−4.754)
*R&D*	0.000 **	0.000 ***
	(2.519)	(3.122)
*LOSS*	−0.001	−0.001 *
	(−1.549)	(−1.902)
*LEV*	−0.000	−0.000
	(−1.011)	(−1.132)
*OPCF*	−0.004 ***	−0.008 ***
	(−4.125)	(−5.550)
*TURN*	0.000	0.000
	(0.869)	(1.136)
*CEO_AGE*	0.000	0.000
	(0.132)	(0.076)
*CEO_COM*	0.000	0.000
	(0.239)	(0.300)
*CEO_TEN*	0.000 ***	0.000 ***
	(2.739)	(3.553)
*CEO_DUA*	−0.000	−0.001
	(−1.248)	(−1.570)
Constant	0.002	0.003
	(0.463)	(0.626)
Week FE	Yes	Yes
Industry FE	Yes	Yes
Province FE	Yes	Yes
Adjusted R^2^	0.003	0.005
Observations	178,805	178,805

*Note*: *, **, *** indicate significance at the 0.10, 0.05, and 0.01 levels, respectively. See Appendix A for definitions and measurements of the variables.

**Table 11:** Continued increasing public health threats and accumulative abnormal return conditioning on the start of community lockdown.

	*CAR* [-1, 1]	*CAR* [−2, 2]
	(A)	(B)
*CIPHT*	−0.007 ***	−0.012 ***
	(−7.105)	(−8.778)
*CIPHT × POST_Lockdown*	0.007 ***	0.011 ***
	(6.766)	(8.379)
*POST_Lockdown*	−0.006 ***	−0.010 ***
	(−6.449)	(−7.826)
*PRO_CASE*	0.000 **	0.001 ***
	(2.319)	(2.945)
*SIZE*	−0.000	−0.000
	(−0.592)	(−0.984)
*ROA*	−0.000	−0.000
	(−0.986)	(−1.196)
*CURR*	−0.000 ***	−0.000 ***
	(−3.666)	(−4.759)
*R&D*	0.000 **	0.000 ***
	(2.519)	(3.122)
*LOSS*	−0.001	−0.001 *
	(−1.549)	(−1.902)
*LEV*	−0.000	−0.000
	(−1.012)	(−1.134)
*OPCF*	−0.004 ***	−0.008 ***
	(−4.130)	(−5.557)
*TURN*	0.000	0.000
	(0.867)	(1.133)
*CEO_AGE*	0.000	0.000
	(0.132)	(0.075)
*CEO_COM*	0.000	0.000
	(0.239)	(0.301)
*CEO_TEN*	0.000 ***	0.000 ***
	(2.744)	(3.558)
*CEO_DUA*	−0.000	−0.001
	(−1.247)	(−1.568)
Constant	0.004	0.008
	(1.164)	(1.620)
Week FE	Yes	Yes
Industry FE	Yes	Yes
Province FE	Yes	Yes
Adjusted R^2^	0.003	0.005
Observations	178,805	178,805

*Note*: *, **, *** indicate significance at the 0.10, 0.05, and 0.01 levels, respectively. See Appendix A for definitions and measurements of the variables.

**Table 12:** Continued increasing public health threats and accumulative abnormal return conditioning on firm-level characteristics.

	*CAR* [−1, 1]	*CAR* [−1, 1]	*CAR* [−1, 1]	*CAR* [−2, 2]	*CAR* [−2, 2]	*CAR* [−2, 2]
	(A)	(B)	(C)	(D)	(E)	(F)
*CIPHT*	−0.002 ***	−0.002 ***	−0.005 ***	−0.003 ***	−0.002 ***	−0.008 ***
	(−4.580)	(−3.633)	(−4.666)	(−5.843)	(−4.384)	(−5.603)
*CIPHT × High_FSALE*	0.004 ***			0.007 ***		
	(7.277)			(9.758)		
*High_FSALE*	−0.003 ***			−0.005 ***		
	(−9.163)			(−11.684)		
*CIPHT × High_OPCF*		0.002 ***			0.004 ***	
		(4.330)			(5.222)	
*High_OPCF*		−0.002 ***			−0.003 ***	
		(−5.731)			(−7.332)	
*CIPHT × Clean_OPIN*			0.004 ***			0.006 ***
			(4.067)			(4.877)
*Clean_OPIN*			−0.001			−0.001
			(−0.887)			(−1.204)
*PRO_CASE*	0.000 ***	0.000 ***	0.000 ***	0.001 ***	0.001 ***	0.001 ***
	(3.070)	(3.074)	(3.058)	(3.891)	(3.894)	(3.875)
*SIZE*	0.000	−0.000	−0.000	0.000	−0.000	−0.000
	(1.476)	(−0.947)	(−0.818)	(1.558)	(−1.458)	(−1.234)
*ROA*	−0.000	−0.000	−0.000	−0.000	−0.000	−0.000
	(−1.004)	(−1.015)	(−1.106)	(−1.219)	(−1.232)	(−1.333)
*CURR*	−0.000 ***	−0.000 ***	−0.000 ***	−0.000 ***	−0.000 ***	−0.000 ***
	(−3.863)	(−3.299)	(−3.775)	(−5.000)	(−4.267)	(−4.879)
*R&D*	0.000 **	0.000 **	0.000 **	0.000 ***	0.000 ***	0.000 ***
	(2.453)	(2.497)	(2.524)	(3.041)	(3.094)	(3.128)
*LOSS*	−0.001	−0.001 *	−0.001	−0.001 *	−0.001 **	−0.001 *
	(−1.454)	(−1.657)	(−1.341)	(−1.787)	(−2.046)	(−1.672)
*LEV*	−0.000	−0.000	−0.000	−0.000	−0.000	−0.000
	(−0.981)	(−1.039)	(−0.831)	(−1.099)	(−1.168)	(−0.940)
*OPCF*	−0.004 ***	−0.002 **	−0.004 ***	−0.008 ***	−0.004 ***	−0.008 ***
	(−4.078)	(−1.998)	(−4.225)	(−5.497)	(−2.712)	(−5.668)
*TURN*	0.000	0.000	0.000	0.000	0.000	0.000
	(1.086)	(0.862)	(0.955)	(1.401)	(1.127)	(1.226)
*CEO_AGE*	−0.000	0.000	0.000	−0.000	0.000	−0.000
	(−0.077)	(0.233)	(0.020)	(−0.183)	(0.210)	(−0.048)
*CEO_COM*	0.000	0.000	0.000	0.000	0.000	0.000
	(0.178)	(0.210)	(0.236)	(0.228)	(0.260)	(0.297)
*CEO_TEN*	0.000 ***	0.000 ***	0.000 ***	0.000 ***	0.000 ***	0.000 ***
	(3.051)	(2.588)	(2.769)	(3.935)	(3.349)	(3.589)
*CEO_DUA*	−0.000	−0.000	−0.000	−0.001	−0.001	−0.001 *
	(−1.177)	(−1.170)	(−1.326)	(−1.484)	(−1.463)	(−1.657)
Constant	−0.002	0.004	0.004	−0.002	0.006	0.007
	(−0.404)	(1.001)	(1.017)	(−0.451)	(1.285)	(1.289)
Week FE	Yes	Yes	Yes	Yes	Yes	Yes
Industry FE	Yes	Yes	Yes	Yes	Yes	Yes
Province FE	Yes	Yes	Yes	Yes	Yes	Yes
Adjusted R^2^	0.003	0.003	0.003	0.006	0.005	0.005
Observations	178,805	178,805	178,805	178,805	178,805	178,805

*Note*: *, **, *** indicate significance at the 0.10, 0.05, and 0.01 levels, respectively. See Appendix A for definitions and measurements of the variables.

**Table 13:** Continued increasing public health threats and accumulative abnormal return conditioning on the volatility of provincial increase in new COVID-19 cases.

	*CAR* [−1, 1]	*CAR* [−2, 2]
	(A)	(B)
*CIPHT*	−0.001 **	−0.002 ***
	(−2.533)	(−3.177)
*CIPHT × High_Volatility*	−0.001	−0.001
	(−1.089)	(−1.022)
*High_Volatility*	0.002 **	0.002 ***
	(2.448)	(2.765)
*PRO_CASE*	0.000 ***	0.001 ***
	(3.114)	(3.930)
*SIZE*	−0.000	−0.000
	(−0.586)	(−0.977)
*ROA*	−0.000	−0.000
	(−0.986)	(−1.196)
*CURR*	−0.000 ***	−0.000 ***
	(−3.672)	(−4.766)
*R&D*	0.000 **	0.000 ***
	(2.519)	(3.122)
*LOSS*	−0.001	−0.001 *
	(−1.549)	(−1.902)
*LEV*	−0.000	−0.000
	(−1.010)	(−1.132)
*OPCF*	−0.004 ***	−0.008 ***
	(−4.126)	(−5.551)
*TURN*	0.000	0.000
	(0.867)	(1.132)
*CEO_AGE*	0.000	0.000
	(0.133)	(0.077)
*CEO_COM*	0.000	0.000
	(0.239)	(0.301)
*CEO_TEN*	0.000 ***	0.000 ***
	(2.742)	(3.556)
*CEO_DUA*	−0.000	−0.001
	(−1.253)	(−1.575)
Constant	0.003	0.004
	(0.663)	(0.862)
Week FE	Yes	Yes
Industry FE	Yes	Yes
Province FE	Yes	Yes
Adjusted R^2^	0.003	0.005
Observations	178,805	178,805

*Note*: *, **, *** indicate significance at the 0.10, 0.05, and 0.01 levels, respectively. See Appendix A for definitions and measurements of the variables.

## Appendix A

**Table A1:** Variable definitions.

Variables	Definitions
*CAR* [−1, 1]	The firm’s three-day cumulative abnormal return [−1, 1] computed by the market model. The market model is estimated over the period [−200, −60] relative to the current day with the market return;
*CAR* [−2, 2]	The firm’s five-day cumulative abnormal return [−2, 2] computed by the market model. The market model is estimated over the period [−200, −60] relative to the current day with the market return;
*CDPHT*	Continued decreasing public health threats that is measured as an indicator variable that equals one if there have not been any provincial new COVID-19 cases for at least six consecutive days including the current day and zero otherwise;
*CEO_AGE*	The age of the firm’s CEO in 2019;
*CEO_COM*	The ratio of the firm’s CEO compensation to the net income in 2019;
*CEO_DUA*	Indicator variable that equals one if the firm’s CEO holds a concurrent post in other work units in 2019 and zero otherwise;
*CEO_TEN*	The tenure of the firm’s CEO that is defined as days of CEO’s tenure in 2019 divided by 365;
*CIPHT*	Continued increasing public health threats that is measured as an indicator variable that equals one if there have been provincial new COVID-19 cases for at least six consecutive days including the current day and zero otherwise;
*CIPHT2*	Alternative variable for continued increasing public health threats that is measured as an indicator variable that equals one if there have been provincial new COVID-19 cases for at least seven consecutive days including the current day and zero otherwise;
*CIPHT3*	Alternative variable for continued increasing public health threats that is measured as an indicator variable that equals one if there have been provincial new COVID-19 cases for at least five consecutive days including the current day and zero otherwise;
*Clean_OPIN*	Indicator variable that equals one if the firm received a clean audit opinion for its financial report in 2019 and zero otherwise;
*CURR*	The current ratio in 2019, measured as a ratio of current assets to current liabilities;
*EMIGRANT*	The six-day mean value (t-5 to t) of the ratio of the daily provincial emigrants to national emigrants (%);
*High_EMR*	Indicator variable that equals one if the provincial employment rate in the urban area (%) in 2019 is higher than or equal to the upper quartile value and zero otherwise;
*High_FSALE*	Indicator variable that equals one if the firm’s foreign sales in 2019 are higher than or equal to the upper quartile value and zero otherwise;
*High_GRP*	Indicator variable that equals one if the provincial ratio of the gross regional product (100 million yuan) to resident population (10,000 persons) in 2019 is higher than or equal to the upper quartile value and zero otherwise;
*High_MED*	Indicator variable that equals one if the provincial TV coverage rate of population in 2019 is higher than or equal to the upper quartile value and zero otherwise;
*High_MOB*	Indicator variable that equals one if the provincial ratio of flow accessed to mobile internet (100 GB) to resident population (10,000 persons) in 2019 is higher than or equal to the upper quartile value and zero otherwise;
*High_OPCF*	Indicator variable that equals one if the ratio of the firm’s operating cash flow to total assets in 2019 is higher than or equal to the upper quartile value and zero otherwise;
*High_URB*	Indicator variable that equals one if the provincial ratio of urban population to the resident population in 2019 is higher than or equal to the upper quartile value and zero otherwise;
*High_Volatility*	Indicator variable that equals one if the six-day standard deviation (t-5 to t) of the new confirmed COVID-19 cases is higher than or equal to the upper quartile value and zero otherwise;
*High_WEB*	Indicator variable that equals one if the provincial ratio of the number of websites per 100 enterprises (unit) to resident population (10,000 persons) in 2019 is higher than or equal to the upper quartile value and zero otherwise;
*IMMIGRANT*	The six-day mean value (t-5 to t) of the ratio of the daily provincial immigrants to the national immigrants (%);
*LEV*	The leverage ratio of total liabilities to total assets in 2019;
*LOSS*	Indicator variable that equals one if the firm suffered a loss in 2019 and zero otherwise;
*OPCF*	The ratio of the firm’s operating cash flow to total assets in 2019;
*POST_Lockdown*	Indicator variable that equals one if the firm is in periods after implementing the "closed community management" measures and zero otherwise;
*PRO_CASE*	The six-day mean value (t-5 to t) of the provincial ratio of the daily accumulated confirmed COVID-19 cases (unit) to resident population (10,000 persons) in 2019;
*Pseudo_CIPHT*	Pseudo continued increasing public health threats that is measured as an indicator variable that equals one if the firm located in the province where faced a continued increase of new COVID-19 cases in the last five days, but no new cases occurred in the current day and zero otherwise;
*R&D*	The ratio of R&D expenses to sales in 2019;
*ROA*	Return on assets in 2019, measured as a ratio of net income to total assets;
*SIZE*	Natural logarithm of total assets in 2019; and
*TURN*	The asset turnover ratio in 2019, measured as the ratio of sales to total assets.

## Appendix B

**Table A2:** Distribution of firm-day observations by province and day.

**Panel A: Distribution of Firm-Day Observations by Province from 10 January to 28 February 2020**																								
	***CIPHT* = 1**																							**Full sample**
	**1/21**	**22**	**23**	**2/03**	**04**	**05**	**06**	**07**	**10**	**11**	**12**	**13**	**14**	**17**	**18**	**19**	**20**	**21**	**24**	**25**	**26**	**27**	**28**	**1/10–2/28**
Anhui	0	0	0	92	92	92	**92**	**92**	**92**	**92**	**92**	**92**	**92**	**92**	**93**	**93**	**93**	**93**	**0**	**0**	**0**	**0**	**0**	2769
Beijing	0	0	0	376	377	377	377	377	377	**377**	**378**	**378**	**378**	**378**	**378**	**378**	**378**	**378**	**0**	**0**	**0**	**0**	**0**	11,325
Chongqing	0	**0**	**0**	**45**	**45**	**45**	**45**	**45**	**45**	**45**	**45**	**45**	**45**	**45**	**45**	**45**	**45**	**45**	**45**	**0**	**0**	**0**	**0**	1350
Fujian	0	0	0	126	126	**126**	**126**	**126**	**126**	**126**	**126**	**126**	**126**	**126**	**126**	**0**	**0**	**0**	**0**	**0**	**0**	**0**	**0**	3780
Gansu	0	0	0	24	24	24	24	24	**24**	**0**	**0**	**0**	**0**	**0**	**0**	**0**	**0**	**0**	**0**	**0**	**0**	**0**	**0**	720
Guangdong	0	0	0	579	580	580	580	**580**	**580**	**580**	**580**	**580**	**580**	**581**	**581**	**581**	**581**	**581**	**582**	**0**	**0**	**0**	**0**	17,376
Guangxi	0	0	0	30	30	30	**30**	**30**	**30**	**30**	**0**	**0**	**0**	**0**	**30**	**30**	**30**	**30**	**0**	**0**	**0**	**0**	**0**	900
Guizhou	0	0	0	25	25	25	25	25	**25**	**25**	**25**	**25**	**25**	**0**	**0**	**0**	**0**	**0**	**0**	**0**	**0**	**0**	**0**	750
Hainan	0	0	0	23	23	23	**23**	**23**	**23**	**23**	**23**	**0**	**0**	**0**	**0**	**0**	**0**	**0**	**0**	**0**	**0**	**0**	**0**	690
Hebei	0	0	0	52	52	52	**52**	**52**	**52**	**52**	**52**	**52**	**52**	**52**	**52**	**52**	**52**	**52**	**0**	**0**	**0**	**0**	**0**	1560
Heilongjiang	0	0	0	28	28	**28**	**28**	**28**	**28**	**28**	**28**	**28**	**28**	**28**	**28**	**28**	**28**	**0**	**0**	**0**	**0**	**0**	**0**	840
Henan	0	0	0	71	71	**71**	**71**	**71**	**71**	**71**	**71**	**71**	**71**	**71**	**71**	**71**	**71**	**71**	**0**	**0**	**0**	**0**	**0**	2122
Hubei	79	79	79	79	79	79	79	79	**79**	**79**	**79**	**79**	**79**	**79**	**79**	**79**	**79**	**79**	**79**	**79**	**79**	**79**	**79**	2368
Hunan	0	0	0	92	92	92	92	92	92	92	92	92	92	92	92	92	92	92	0	0	0	0	0	2751
Jiangsu	0	0	0	383	**384**	**384**	**384**	**384**	**384**	**384**	**384**	**384**	**384**	**384**	**384**	**0**	**0**	**0**	**0**	**0**	**0**	**0**	**0**	11,500
Jiangxi	0	0	0	41	41	**41**	**41**	**41**	**41**	**41**	**41**	**41**	**41**	**41**	**40**	**41**	**0**	**0**	**0**	**0**	**0**	**0**	**0**	1229
Jilin	0	0	0	0	0	29	29	**29**	**29**	**29**	**29**	**29**	**29**	**0**	**0**	**0**	**0**	**0**	**0**	**0**	**0**	**0**	**0**	865
Liaoning	0	0	0	66	66	66	66	**66**	**66**	**66**	**0**	**0**	**0**	**0**	**0**	**0**	**0**	**0**	**0**	**0**	**0**	**0**	**0**	1980
Neimenggu	0	0	0	20	20	20	20	20	0	0	0	**0**	**0**	**20**	**20**	**0**	**0**	**0**	**0**	**0**	**0**	**0**	**0**	600
Ningxia	0	**0**	**0**	**14**	**0**	**0**	**0**	**0**	**14**	**14**	**14**	**14**	**14**	**0**	**0**	**0**	**0**	**0**	**0**	**0**	**0**	**0**	**0**	420
Qinghai	0	0	0	0	0	0	0	0	0	0	0	0	0	0	0	0	0	0	0	0	0	0	0	299
Shaanxi	0	0	0	46	46	47	47	47	**47**	**47**	**47**	**47**	**47**	**47**	**0**	**0**	**0**	**0**	**0**	**0**	**0**	**0**	**0**	1395
Shandong	0	0	0	185	185	**185**	**185**	**185**	**185**	**185**	**185**	**185**	**185**	**185**	**185**	**185**	**185**	**185**	**0**	**0**	**0**	**0**	**0**	5553
Shanghai	0	0	0	312	312	312	312	312	312	**313**	**314**	**314**	**314**	**314**	**0**	**0**	**0**	**0**	**0**	**0**	**0**	**0**	**0**	9393
Shanxi	0	0	0	31	31	31	31	31	31	31	31	0	0	0	0	0	0	0	0	0	0	0	0	930
Sichuan	0	0	0	115	115	115	115	**115**	**115**	**115**	**115**	**115**	**115**	**115**	**115**	**115**	**115**	**115**	**0**	**0**	**0**	**0**	**0**	3445
Tianjin	0	0	0	42	42	42	42	42	**42**	**42**	**42**	**42**	**42**	**42**	**42**	**42**	**42**	**42**	**0**	**0**	**0**	**0**	**0**	1262
Xinjiang	0	0	0	45	45	45	45	45	45	45	45	45	45	45	0	0	0	0	0	0	0	0	0	1347
Xizang	0	0	0	0	0	0	0	0	0	0	0	0	0	0	0	0	0	0	0	0	0	0	0	270
Yunnan	0	0	0	31	31	31	**31**	**31**	**31**	**31**	**30**	**30**	**31**	**31**	**31**	**0**	**0**	**0**	**0**	**0**	**0**	**0**	**0**	928
Zhejiang	0	0	0	**403**	**403**	**403**	**403**	**403**	**403**	**403**	**403**	**403**	**403**	**403**	**403**	**403**	**403**	**403**	**0**	**0**	**0**	**0**	**0**	12,075
**Panel B: Distribution of Firm-Day Observations by Province from 2 March to 31 March 2020**																								
		***CIPHT* = 1**																						**Full sample**
	**3/02**	**03**	**04**	**05**	**06**	**09**	**10**	**11**	**12**	**13**	**16**	**17**	**18**	**19**	**20**	**23**	**24**	**25**	**26**	**27**	**30**	**31**		**3/02–3/31**
Anhui	**0**	**0**	**0**	**0**	**0**	**0**	**0**	**0**	**0**	**0**	**0**	**0**	**0**	**0**	**0**	**0**	**0**	**0**	**0**	**0**	**0**	**0**		2047
Beijing	**0**	**0**	**0**	**0**	**0**	**0**	**0**	**0**	**0**	**0**	**0**	**385**	**384**	**383**	**384**	**385**	**385**	**385**	**385**	**385**	**384**	**0**		8435
Chongqing	**0**	**0**	**0**	**0**	**0**	**0**	**0**	**0**	**0**	**0**	**0**	**0**	**0**	**0**	**0**	**0**	**0**	**0**	**0**	**0**	**0**	**0**		989
Fujian	**0**	**0**	**0**	**0**	**0**	**0**	**0**	**0**	**0**	**0**	**0**	**0**	**0**	**0**	**0**	**0**	**128**	**128**	**128**	**128**	**128**	**128**		2792
Gansu	**0**	**0**	**0**	**0**	**0**	**0**	**0**	**0**	**0**	**0**	**0**	**0**	**0**	**0**	**0**	**0**	**0**	**0**	**0**	**0**	**0**	**0**		527
Guangdong	**0**	**0**	**0**	**0**	**0**	**0**	**0**	**0**	**0**	**0**	**0**	**0**	**0**	**587**	**588**	**588**	**589**	**589**	**589**	**589**	**590**	**591**		12,893
Guangxi	**0**	**0**	**0**	**0**	**0**	**0**	**0**	**0**	**0**	**0**	**0**	**0**	**0**	**0**	**0**	**0**	**0**	**0**	**0**	**0**	**0**	**0**		650
Guizhou	**0**	**0**	**0**	**0**	**0**	**0**	**0**	**0**	**0**	**0**	**0**	**0**	**0**	**0**	**0**	**0**	**0**	**0**	**0**	**0**	**0**	**0**		550
Hainan	**0**	**0**	**0**	**0**	**0**	**0**	**0**	**0**	**0**	**0**	**0**	**0**	**0**	**0**	**0**	**0**	**0**	**0**	**0**	**0**	**0**	**0**		505
Hebei	**0**	**0**	**0**	**0**	**0**	**0**	**0**	**0**	**0**	**0**	**0**	**0**	**0**	**0**	**0**	**0**	**0**	**0**	**0**	**0**	**0**	**0**		1144
Heilongjiang	**0**	**0**	**0**	**0**	**0**	**0**	**0**	**0**	**0**	**0**	**0**	**0**	**0**	**0**	**0**	**0**	**0**	**0**	**0**	**0**	**0**	**0**		595
Henan	**0**	**0**	**0**	**0**	**0**	**0**	**0**	**0**	**0**	**0**	**0**	**0**	**0**	**0**	**0**	**0**	**0**	**0**	**0**	**0**	**0**	**0**		1562
Hubei	**79**	**79**	**79**	**79**	**79**	**79**	**79**	**79**	**79**	**79**	**79**	**79**	**0**	**0**	**0**	**0**	**0**	**0**	**0**	**0**	**0**	**0**		1730
Hunan	0	0	0	0	0	0	0	0	0	0	0	0	0	0	0	0	0	0	0	0	0	0		2032
Jiangsu	**0**	**0**	**0**	**0**	**0**	**0**	**0**	**0**	**0**	**0**	**0**	**0**	**0**	**0**	**0**	**0**	**0**	**0**	**0**	**0**	**0**	**0**		8494
Jiangxi	**0**	**0**	**0**	**0**	**0**	**0**	**0**	**0**	**0**	**0**	**0**	**0**	**0**	**0**	**0**	**0**	**0**	**0**	**0**	**0**	**0**	**0**		892
Jilin	**0**	**0**	**0**	**0**	**0**	**0**	**0**	**0**	**0**	**0**	**0**	**0**	**0**	**0**	**0**	**0**	**0**	**0**	**0**	**0**	**0**	**0**		638
Liaoning	**0**	**0**	**0**	**0**	**0**	**0**	**0**	**0**	**0**	**0**	**0**	**0**	**0**	**0**	**0**	**0**	**0**	**0**	**0**	**0**	**0**	**66**		1440
Neimenggu	**0**	**0**	**0**	**0**	**0**	**0**	**0**	**0**	**0**	**0**	**0**	**0**	**0**	**0**	**0**	**0**	**0**	**0**	**0**	**0**	**20**	**20**		440
Ningxia	**0**	**0**	**0**	**0**	**0**	**0**	**0**	**0**	**0**	**0**	**0**	**0**	**0**	**0**	**0**	**0**	**0**	**0**	**0**	**0**	**0**	**0**		308
Qinghai	**0**	**0**	**0**	**0**	**0**	**0**	**0**	**0**	**0**	**0**	**0**	**0**	**0**	**0**	**0**	**0**	**0**	**0**	**0**	**0**	**0**	**0**		220
Shaanxi	**0**	**0**	**0**	**0**	**0**	**0**	**0**	**0**	**0**	**0**	**0**	**0**	**0**	**0**	**0**	**0**	**0**	**0**	**0**	**0**	**0**	**0**		1034
Shandong	**0**	**0**	**0**	**0**	**0**	**0**	**0**	**0**	**0**	**0**	**0**	**0**	**0**	**0**	**0**	**0**	**187**	**0**	**0**	**0**	**0**	**0**		4117
Shanghai	**0**	**0**	**0**	**0**	**0**	**0**	**0**	**0**	**0**	**0**	**0**	**319**	**319**	**319**	**318**	**318**	**318**	**317**	**318**	**318**	**318**	**318**		6996
Shanxi	0	0	0	0	0	0	0	0	0	0	0	0	0	0	0	0	0	0	0	0	0	0		682
Sichuan	**0**	**0**	**0**	**0**	**0**	**0**	**0**	**0**	**0**	**0**	**0**	**0**	**0**	**0**	**0**	**0**	**0**	**0**	**0**	**0**	**0**	**0**		2529
Tianjin	**0**	**0**	**0**	**0**	**0**	**0**	**0**	**0**	**0**	**0**	**0**	**0**	**0**	**0**	**0**	**0**	**0**	**0**	**0**	**0**	**44**	**0**		966
Xinjiang	0	0	0	0	0	0	0	0	0	0	0	0	0	0	0	0	0	0	0	0	0	0		988
Xizang	0	0	0	0	0	0	0	0	0	0	0	0	0	0	0	0	0	0	0	0	0	0		198
Yunnan	**0**	**0**	**0**	**0**	**0**	**0**	**0**	**0**	**0**	**0**	**0**	**0**	**0**	**0**	**0**	**0**	**0**	**0**	**0**	**0**	**0**	**0**		682
Zhejiang	**0**	**0**	**0**	**0**	**0**	**0**	**0**	**0**	**0**	**0**	**0**	**0**	**0**	**0**	**0**	**406**	**406**	**406**	**407**	**407**	**408**	**0**		8938

*Note*: Values in bold are in periods after implementing the “closed community management” measures.
